# Supplementary material for: The African Swine Fever Epidemic in Wild Boar (Sus scrofa) in Lithuania (2014–2018)
Source: Vet Sci. 2020 Jan 30;7(1):15. doi: 10.3390/vetsci7010015 (PMC7157679; doi:10.3390/vetsci7010015)
Supplement: Supplementary file 1 [file vetsci-07-00015-s001.pdf]

Supplementary Information

Supplementary Table S1. Overview of laboratory results of wild boar samples obtained in Lithuania during 2014-2018

Year	Wild boar found dead		Wild boar hunted			
	Tested	PCR positive (%)	Tested	PCR positive (%)	Tested	Antibody positive (%)
2014	241	43 (17.8)	14981	26 (0.17)	7863	1 (0.01)
2015	246	65 (26.4)	12590	37 (0.29)	7219	23 (0.32)
2016	734	375 (51.1)	17115	50 (0.29)	14660	57 (0.39)
2017	2751	2083 (75.7)	16043	247 (1.54)	13917	98 (0.70)
2018	2248	1769 (78.7)	14131	225 (1.59)	11358	190 (1.67)
**Total**	**6220**	**4335 (69.7)**	**74860**	**585 (0.78)**	**55017**	**369 (0.67)**

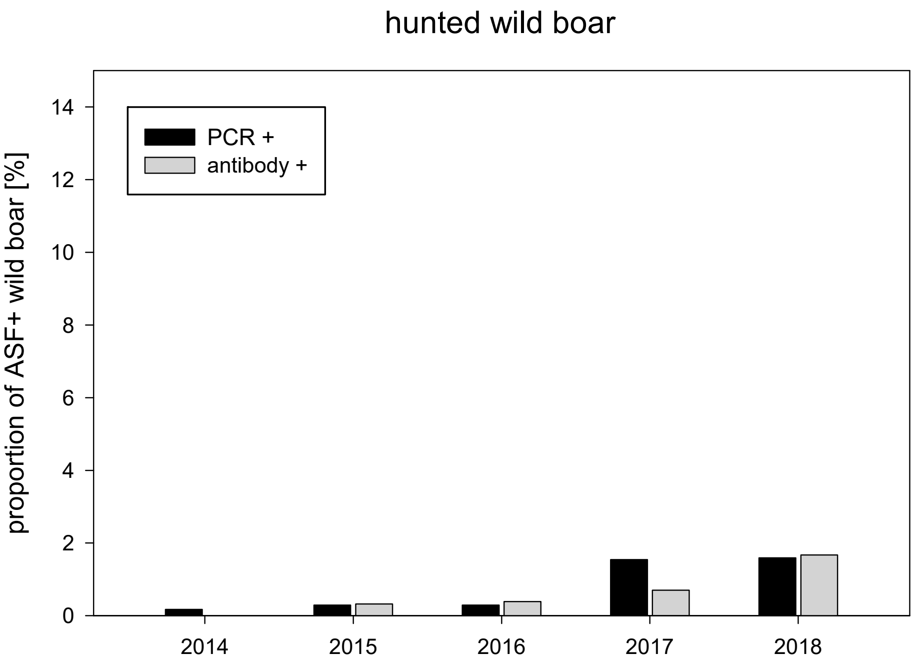

Supplementary Figure S1. Proportion of PCR+ and antibody+ wild boar samples

Supplementary Table S2. Overview of laboratory results of wild boar samples by seasons in Lithuania (2014-2018)

Year		Found dead		Hunted			
		Tested	PCR positive (%)	Tested	PCR positive (%)	Tested	Antibody positive (%)
2014	spring	42	0 (0)	5207	0 (0)	2699	0 (0)
	summer	52	1 (1.9)	910	0 (0)	1354	0 (0)
	autumn	106	27 (25.5)	1504	9 (0.60)	882	1 (0.11)
	winter	41	15 (36.6)	7360	17 (0.23)	2928	0 (0)
2015	spring	83	28 (33.7)	1643	1 (0.06)	991	1 (0.10)
	summer	48	14 (29.2)	2993	7 (0.23)	2109	4 (0.19)
	autumn	60	8 (13.3)	4175	17 (0.41)	2465	12 (0.49)

	winter	55	15 (27.3)	3779	12 (0.32)	1654	6 (0.36)
2016	spring	191	83 (43.5)	3145	4 (0.13)	2843	3 (0.11)
	summer	297	164 (55.2)	3841	16 (0.42)	3821	11 (0.29)
	autumn	140	70 (50.0)	4009	13 (0.32)	3931	28 (0.71)
	winter	106	58 (54.7)	6120	17 (0.28)	4065	15 (0.37)
2017	spring	359	186 (51.8)	2777	8 (0.29%)	2408	4 (0.17)
	summer	669	475 (70.9)	4145	58 (1.40%)	3752	17 (0.45)
	autumn	1031	842 (81.7)	3709	101 (2.72%)	3089	42 (1.36)
	winter	692	610 (87.9)	5412	65 (1.20%)	4668	35 (0.75)
2018	spring	805	624 (77.5)	2989	19 (0.64%)	2510	25 (1.00)
	summer	288	187 (64.9)	2861	50 (1.75%)	2529	35 (1.38)
	autumn	172	94 (54.7)	2998	31 (1.03%)	2356	61 (2.59)
	winter	983	864 (87.9)	5283	125 (2.36%)	3963	69 (1.74)

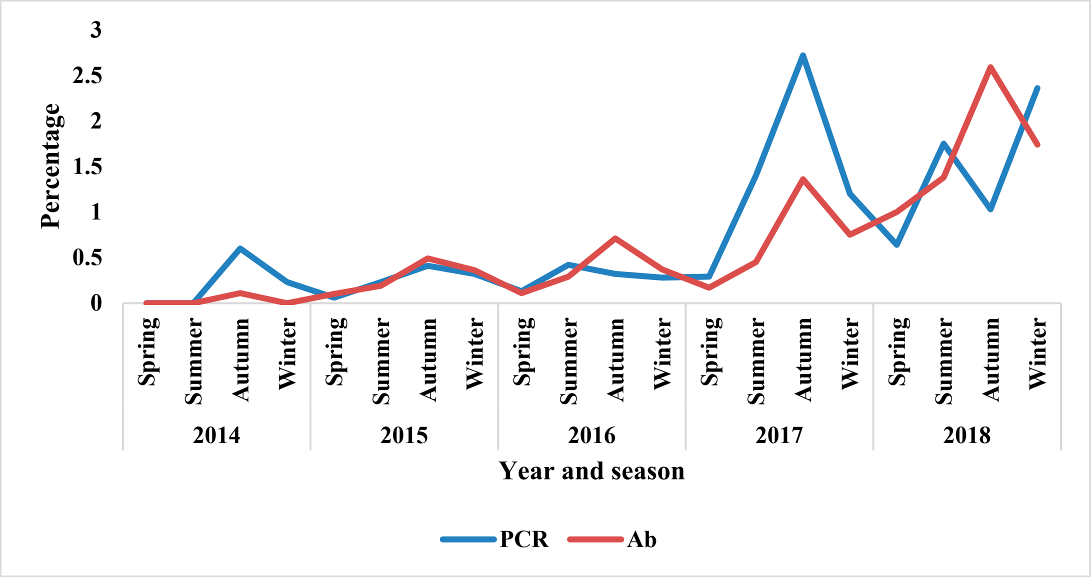

Supplementary Figure S2. Proportion of PCR and antibody positive hunted wild boar during the seasons 2014-2018

Supplementary Table S3. Number municipalities and elderships affected by ASF during 2014-2018

Year	Positive municipalities	New affected municipalities	Positive eldership	New affected eldership
2014	11	-	25	-
2015	18	8	57	45
2016	19	3	66	35
2017	30	10	157	101
2018	41	10	237	94
Total	42		300	

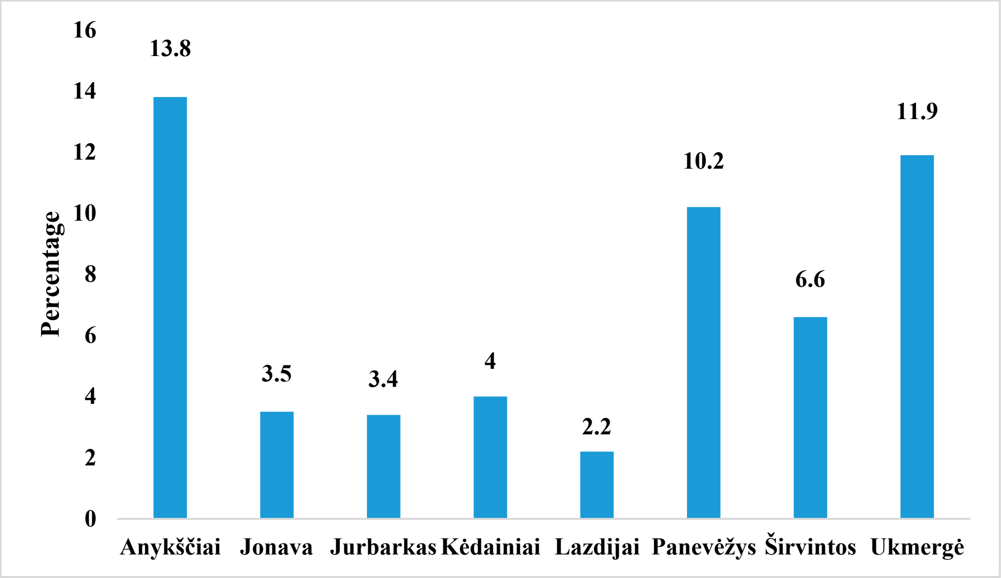

Supplementary Figure S3. Proportion of ASF cases in wild boar on municipality level (2014–2018)
